# Use of Continuous Renal Replacement Therapy for Removal of Dabigatran in a Patient in Need of Emergent Surgery

**DOI:** 10.1155/2016/9692568

**Published:** 2016-05-26

**Authors:** Sara E. Parli, Melissa L. Thompson Bastin, Daniel A. Lewis

**Affiliations:** ^1^Department of Pharmacy Services, University of Kentucky HealthCare, Lexington, KY 40536, USA; ^2^Department of Pharmacy Practice and Science, University of Kentucky College of Pharmacy, Lexington, KY 40536, USA; ^3^Department of Pharmacy Services, South Pointe Hospital, Cleveland Clinic, Cleveland, OH 44195, USA

## Abstract

*Purpose.* To report the ability to remove serum dabigatran using continuous renal replacement therapy (CRRT) in a patient with life-threatening bleeding.* Summary.* A 77-year-old female with history of atrial fibrillation who takes dabigatran for stroke prevention presented with abdominal pain. Patient was found to have bleeding and possible mesenteric ischemia and was taken to the operating room and had continued bleeding postoperatively. CRRT was initiated for the removal of any remaining dabigatran, with serum dabigatran levels collected to evaluate removal of dabigatran with CRRT. This patient had an increased dabigatran level prior to intervention, which decreased to an undetectable level after use of CRRT. Greater than 80% of the drug was removed due to 4 hours of CRRT and residual kidney function. Reversal of dabigatran is an area of current research with recent FDA approval of idarucizumab for use.* Conclusion.* Bleeding may occur as a result of the use of dabigatran and change in patient's clinical condition. Use of CRRT may be an option in removing serum dabigatran in the case of a life-threatening bleed.

## 1. Introduction

Dabigatran etexilate is an oral reversible direct thrombin inhibitor, approved for anticoagulation for prevention of stroke and systemic embolism in nonvalvular atrial fibrillation, as well as the treatment and reduction in risk of recurrence of pulmonary embolism and deep vein thrombosis (DVT). It offers a predictable anticoagulation response without the need for routine coagulation test monitoring and therefore is becoming a viable alternative to vitamin K antagonists, such as warfarin [1]. The pharmacokinetics have previously been described in detail [1, 2]. Dabigatran etexilate is rapidly absorbed from the gastrointestinal tract and quickly hydrolyzed to its active form (dabigatran) and follows first-order kinetics with a two-compartment distribution model. Effects of food and drug interactions are negligible, as the anticoagulant effect is immediate and predictable. Metabolism occurs by way of liver conjugation with glucuronic acid, with 80% of unchanged drug recovered in the urine; therefore, dabigatran must be dose-adjusted for renal insufficiency. Steady state concentrations are reached within three days of oral dosing, and the average half-life is 12–17 hours. Although dabigatran exhibits an ideal pharmacokinetic profile, a potential pitfall to its use is the difficulty in accurately assessing the degree of anticoagulation in a supratherapeutic dosing situation [2]. In this report, we describe a case of utilizing continuous renal replacement therapy (CRRT) for the removal of dabigatran from the serum of a patient with an ischemic bowel requiring emergent surgery. The report of this case did not require Institutional Review Board review by the investigators institution.

## 2. Case

A 77-year-old Caucasian female presented to an outside hospital via Emergency Medical Services with complaints of abdominal pain, bright red blood in her stools, and an episode of melena. She was found to be hypotensive and tachycardic and have coagulation lab abnormalities. The international normalized ratio (INR) was elevated at 9 and guaiac test was positive. The patient was given two liters of intravenous fluids, which stabilized her blood pressure before she was transferred to our institution for further evaluation and treatment.

The patient's past medical history included paroxysmal atrial fibrillation, chronic obstructive pulmonary disease, and depression, with past surgical history of hip repair and left above-knee amputation. Pertinent oral home medications included dabigatran etexilate 150 mg twice daily, duloxetine HCl 30 mg every morning and 60 mg at bedtime, carvedilol 6.25 mg daily, lisinopril 2.5 mg daily, furosemide 40 mg daily, and diltiazem 120 mg daily. The last known dose of dabigatran was taken the morning prior to transfer.

Upon admission, the patient was volume-resuscitated with crystalloid fluids, which improved the blood pressure and heart rate. Lab abnormalities on presentation to our facility included hemoglobin and hematocrit (H/H) of 7.2 and 20.6, an increased serum creatinine (SCr) of 1.54 mg/dL (estimated creatinine clearance of 38 mL/min using Cockcroft Gault (CG) formula [weight 80.6 kg]), activated partial thromboplastin time (aPTT) of >160 seconds, INR of 6.2, prothrombin time (PT) of 58.2 seconds, and lactate of 3.5 mmol/L, which decreased to INR of 3.2, prothrombin time of 31.5 seconds, and lactate of 2.4 mmol/L after 2 units of packed red blood cells and 3 units of fresh frozen plasma. Her H/H improved to 9.8 and 27.7, respectively. A physical examination found the patient to have mild abdominal distention with positive bowel sounds and a left lower quadrant implanted pain pump.

**Figure 1:**
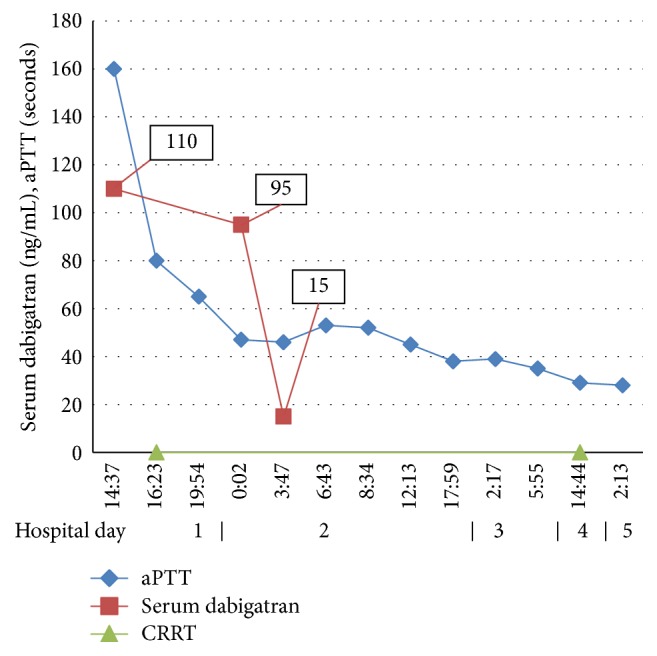
Serum dabigatran levels and aPTT trend over time.

An additional 2 units of packed red blood cells and 3 units of fresh frozen plasma were given due to elevated INR, and the patient was started on a pantoprazole drip for further management of a gastrointestinal bleed as well as antibiotics for possible infection. A computed tomography scan of her abdomen/pelvis was obtained which revealed some thickening of the colon, pneumatosis, and portal venous air, following which she was seen by general surgery and taken emergently to the operating room for an exploratory laparotomy. Ischemia of the hepatic flexure was found and resected. The patient was noted to have a small tear in the liver capsule, which was sprayed with thrombin and Surgicel, covered with laparotomy pads and a wound vacuum assisted closure device was placed. Her labs at this time included H/H of 9.3 and 26.8, INR of 2.8, and aPTT of 80 seconds. Patient had an estimated blood loss of 2 liters in the operating room and continued to have oozing from her wound postoperatively. A nephrology consult was then obtained to initiate dialysis in an attempt to remove any remaining dabigatran from the serum that may be contributing to her current bleeding. Access was obtained, and since the patient remained hypotensive postoperatively despite volume resuscitation with colloids, crystalloids, and norepinephrine infusion, the decision was made to initiate CRRT for treatment of the acute nonoliguric renal failure as well as to augment dabigatran removal from the serum. Serum dabigatran levels were determined using high performance liquid chromatography (HPLC); margin of error was not reported. Samples were collected prior to initiation of CRRT, as well as approximately 8, 12, and 24 hours after initiation of CRRT (Figure 1). Serum drug levels were send-out tests performed at National Medical Services, Willow Grove, PA.

CRRT was initiated as continuous venovenous hemodiafiltration (CVVHD/F). The dialysis set utilized the Prismasate® system, with the HF1400 polyarylethersulfone filter (Gambro®). CVVHD/F hemofiltration was prescribed at a total hemofiltration dose of 2500 ml/hr (32 ml/kg/hr) with a blood flow rate of 250 ml/min, dialysate flow rate of 1500 ml/hr, and replacement flow rate of 1000 mL/hr, with infused 100% post-filter; the preceding settings are common rates used at our institution. Circuit anticoagulation was not initiated as the patient was actively bleeding, requiring transfusions, and extent of liver injury previously described was not yet known. A hemofiltration fluid consisting of bicarbonate 22 mEq/L, calcium 0 mEq/L, chloride 120.5 mEq/L, lactate 3 mEq/L, magnesium 1.5 mEq/L, sodium 140 mEq/L, and potassium 4 mEq/L was utilized for both dialysate and replacement.

The initial dabigatran level, collected approximately 2 hours prior to start of CVVHD/F, was 110 ng/mL. This decreased to 95 ng/mL and 15 ng/mL, 7.5 h (after 5.5 h of CVVHD/F) and 11.5 h (after 9.5 h of CVVHD/F) after initial level, respectively. The final serum level was obtained approximately 24 h after the first level and returned undetectable (after 22 h of CVVHD/F). Dialysis was no longer necessary at this point for the indication of drug toxicity and rather was continued for volume status and hemodynamic instability. In relation to the last known time of ingestion (which was approximately 0900 the morning of transfer), the levels were drawn at the following intervals: 31.5 h, 39 h, 43 h, and 55 h after the dose. The calculated estimated creatinine clearance just prior to initiation of CVVHD/F had improved to 58 mL/min (CG), with adequate urine output of 1 mL/kg/hour which was maintained throughout the first 48 hours of CRRT therapy, as renal function improved daily upon admission. CVVHD/F was continued overnight for approximately 18 hours until the patient was taken back to the operating room on hospital day 2 for relaparotomy and removal of packing and evaluation for ongoing bleeding. She received another unit of packed red blood cells prior to operation, with hemoglobin and hematocrit of 7.4 g/dL and 20.3%, respectively, which was decreased from previous value, as well as INR of 1.6 and aPTT of 53 seconds at the time. Hemorrhage was noted to be controlled, blood and coagulation products were no longer necessary, and the patient underwent resection of nonviable cecum and ileostomy creation. The patient returned to the intensive care unit where CVVHD/F was restarted and continued through hospital day 4 for management of volume status (3 days total of CRRT), at which time she returned to the operating room for removal of packing with fascia and abdominal wall closure. The patient was transferred to a progressive care unit and discharged to a nursing and rehabilitation facility on hospital day 21, a prolonged stay which was not deemed to be related to bleeding complications. She did return to the hospital 4 days after discharge for possible infection requiring an 18-day hospital stay until she was discharged alive.

Pharmacokinetic calculations were done utilizing linear noncompartmental pharmacokinetic methods [3]. The elimination rate constant (*k*_*e*_) was calculated using the only 2 detectable levels which were drawn during the treatment with CRRT (level 2 = 95 ng/mL and level 3 = 15 ng/mL). The final level drawn 22 h after CRRT commencement was not able to be used as it is below the limit of detection of the laboratory. The *k*_*e*_ calculation includes elimination on CRRT plus renal elimination from a calculated GFR of 58 mL/min. Pharmacokinetic parameters are as follows: [*k*_*e*_ = 0.461 hours^−1^] and [elimination half-life = 1.5 hours]. *C*_max_ and *V*_*d*_ were not calculated, as the elimination prior to starting CRRT is not known. Based on calculated clearance, the dabigatran level would be expected to fall below 1 ng/mL approximately 6 hours after the last measured serum concentration. Compared to published pharmacokinetic information, the half-life exhibited during the treatment with CRRT was significantly shorter compared to healthy elderly female volunteers with normal renal function (1.5 hours versus 13 hours) [2] although our patient did have adequate renal function during the treatment with CRRT, and this seems to be a significant decrease in the half-life of this drug. It is not possible to determine exactly how much clearance is due to the effects of the CRRT, as effluent levels were not measured; however, our observed elimination half-life was approximately 8 times faster than expected [2]. Limitations to the pharmacokinetic calculations include assumption of steady state dosing, consistent renal elimination over the time course of serum sampling, compliance with home medication regimen, and inability to calculate *V*_*d*_. Regardless of the inherent limitations of the calculations and serum measurements, we believe that the enhanced clearance seen with CRRT as well as residual renal function is worth noting. While it is impossible to predict the exact elimination from the CRRT circuit without measuring drug effluent levels, the total dabigatran clearance was observed to be significantly higher in our patient, as compared to expected clearance seen in previous pharmacokinetic studies in a similar patient population.

## 3. Discussion

We describe a case of dabigatran induced life-threatening bleeding, a unique case in which the primary treatment modality used for reversal was CRRT. Other cases utilizing CRRT to remove dabigatran from the serum of bleeding patients have been reported [4, 5], yet data are still lacking for removal constants and dosage adjustments during CRRT. Dosing adjustment recommendations exist for patients with moderate renal dysfunction; however, patients with severe renal dysfunction were excluded from phase III clinical trials. A group of patients with end-stage renal disease requiring intermittent hemodialysis were included in a pharmacokinetic study of dabigatran and it was determined that approximately 68% of the 50 mg dose was removed after the 4-hour dialysis session, with 62% removed at 2 hours [6]. Chang et al. describe a case of intermittent hemodialysis for emergent dabigatran removal which resulted in a serum decrease in serum dabigatran concentration of about 10 ng/mL per hour during the time of monitoring. After hemodialysis was completed, further serum monitoring showed a rebound of drug levels possibly due to the large volume of distribution of dabigatran [7]. A well designed pharmacokinetic study by Khadzhynov et al. reports the results of phase I study of dabigatran elimination by hemodialysis [8]. Serum levels of dabigatran were reduced by 59.3% after a four-hour session of hemodialysis at a blood flow rate of 400 mL/min. At a lower blood flow rate of 200 mL/min, they observed a 48.8% removal of serum dabigatran. This study is unique in that the investigators were able to observe greater extent of extracorporeal removal of dabigatran with a higher blood flow rate, although the authors state that this effect was not linear. The investigators of this study did not detect a significant rebound effect of dabigatran after the dialysis session, although clinically significant bleeding due to rebound drug levels has been described in a setting of extracorporeal drug removal [5]. In our case, we did not collect serum dabigatran levels after the termination of CRRT in order to measure a rebound effect. The patient required CRRT for 3 days total to treat the acute nonoliguric renal failure and dabigatran toxicity; it was assumed at that time that the dabigatran had been adequately removed from the serum, renal function had recovered, and the clinically significant bleeding had since stopped.

Reversal of dabigatran is difficult due to its specific action on thrombin generation. Vitamin K and protamine will have little to no effect on reversal [9]. Use of recombinant factors has been investigated as potential reversal agents, including recombinant factor VIIa which has been used to study the reversal effects of direct thrombin inhibitors with conflicting results [7, 10]. Rat models of bleeding have shown that activated factor products (PCC), such as FEIBA® (Factor Eight Inhibitor Bypassing Activity), have significantly slowed bleeding times following dabigatran overdose [11]. In contrast, nonactivated PCCs, such as Cofact®, have not had the same reversal effects on anticoagulation due to dabigatran [11]. Additional limitations include the lack of routinely available four-factor PCCs in the United States at some institutions due to cost constraints. In an ex vivo model of dabigatran therapy, Khoo and colleagues examined the effects of FEIBA on coagulation abnormalities induced by dabigatran at therapeutic doses (serum concentration range 96–286 ng/mL). The authors found that a 50 u/kg dose was efficacious in reversing the effects of dabigatran [12]. A case presented by Dager et al. reported successful reversal of a life-threatening bleed caused by dabigatran utilizing FEIBA [13]. A dose of 26 u/kg actual body weight was used to treat the bleeding caused by a transseptal perforation in the presence of anticoagulation with dabigatran. The aPTT values did not correlate well with clinical bleeding or doses of FEIBA; however, the bleeding time decreased in a linear manner after administration of the FEIBA [12, 13]. Idarucizumab, a monoclonal antibody fragment, binds to both free and thrombin-bound dabigatran to inhibit its activity and recently gained fast track FDA approval. The RE-VERSE AD trial showed 100% median maximum percentage reversal in patients taking dabigatran who had serious bleeding or required a procedure within 8 hours [14]. Given the lack of clinical experience of the product, safety concerns exist. The RE-VERSE AD trial lists several early and late adverse events of concern including DVT and ischemic stroke; however, none of these patients were receiving antithrombotic therapy in a patient population with known risk (hence taking dabigatran prior to admission). In addition, cost may be an issue as hospitals predict use and inventory management. Critical access hospitals should attempt to carry this product if possible. CRRT may be considered in a patient who is not able to receive this reversal agent given drug acquisition due to unavailability or cost constraints. Additional postmarketing surveillance data will help determine the role of CRRT in these cases.

Dabigatran was developed as an alternative to warfarin with less need for routine laboratory monitoring. There are times when monitoring of the coagulation levels may become necessary, as in the example above during a life-threatening bleeding event, although consensus on a readily available test to define coagulation status in these patients is lacking. Previous studies have shown that the aPTT is prolonged with increasing doses of dabigatran, although the result becomes less accurate with larger doses of the drug [2]. PT and INR are not significantly altered by therapeutic doses of dabigatran (although they can be elevated with supratherapeutic concentrations). The ecarin clotting time (ECT) and thrombin clotting time (TT) have the strongest correlation with increasing serum drug concentrations, although they are not widely available as a point-of-care test in most hospitals [2, 15].

## 4. Conclusion

The case above reports a successful treatment utilizing CRRT to remove dabigatran from the serum of a patient with a life-threatening bleed. The use of CRRT along with residual kidney function removed 84 percent of the drug serum level over approximately 4 hours, enhancing the elimination of the drug 8-fold compared to healthy volunteers. Although a 4-hour intermittent hemodialysis session may be a more efficient modality to dialyze toxic dabigatran levels [5, 8], our reported case enhances the body of literature suggesting that CRRT is a safe and efficacious therapy to employ in the treatment of a dabigatran induced life-threatening bleed in a hemodynamically unstable patient.
